# Supplementary material for: Expression status and clinical significance of lncRNA APPAT in the progression of atherosclerosis
Source: PeerJ. 2018 Jan 17;6:e4246. doi: 10.7717/peerj.4246 (PMC5775756; doi:10.7717/peerj.4246)
Supplement: Table S1 [file peerj-06-4246-s001.docx]

| **Transcript_id** | **Gene_id** | **Length** | **log2(Foldchange)** | **p-value** | **FDR(q-value)** |
| --- | --- | --- | --- | --- | --- |
| TCONS_01701109 | XLOC_837642 | 2823 | 2.27558 | 5.00E-05 | 0.00262727 |
| TCONS_02288701 | XLOC_1125192 | 1841 | 1.95925 | 0.0001 | 0.00483418 |
| TCONS_02636927 | XLOC_1303487 | 432 | 1.80597 | 0.0003 | 0.01173 |
| TCONS_00846803 | XLOC_429164 | 420 | 1.60448 | 5.00E-05 | 0.00262727 |
| TCONS_02499900 | XLOC_1213768 | 1125 | 1.49805 | 5.00E-05 | 0.00262727 |
| TCONS_02236116 | XLOC_1102749 | 981 | 1.13681 | 0.0015 | 0.042609 |
| TCONS_02225105 | XLOC_1096881 | 1587 | 1.02515 | 0.00065 | 0.0216027 |
| TCONS_00469510 | XLOC_258086 | 711 | 0.917575 | 0.00165 | 0.045227 |
| TCONS_00313387 | XLOC_164005 | 650 | 0.871039 | 0.0006 | 0.020348 |
| TCONS_02508911 | XLOC_1218012 | 595 | 0.845144 | 0.00085 | 0.0271632 |
| TCONS_02236106 | XLOC_1102705 | 1348 | 0.837711 | 0.00155 | 0.0433804 |
| TCONS_00353032 | XLOC_206946 | 2732 | 0.828453 | 0.0011 | 0.033235 |
| TCONS_00473436 | XLOC_267328 | 1225 | 0.794316 | 5.00E-05 | 0.00262727 |
| TCONS_01113159 | XLOC_551577 | 695 | 0.729293 | 0.001 | 0.0310607 |
| TCONS_02450577 | XLOC_1192492 | 323 | 0.718435 | 0.0014 | 0.0401978 |
| TCONS_01415027 | XLOC_745984 | 371 | 0.70208 | 0.00035 | 0.0132185 |
| TCONS_02217210 | XLOC_1094003 | 762 | 0.651913 | 0.00105 | 0.0321629 |
| TCONS_01447402 | XLOC_699737 | 3695 | -0.620309 | 0.0009 | 0.0284195 |
| TCONS_00941286 | XLOC_475583 | 3634 | -0.637834 | 0.0006 | 0.020348 |
| TCONS_00250584 | XLOC_127081 | 4129 | -0.787889 | 0.00025 | 0.0100712 |
| TCONS_02469564 | XLOC_1201167 | 385 | -0.822339 | 0.0002 | 0.00857677 |
| TCONS_02092269 | XLOC_1035367 | 953 | -0.866417 | 5.00E-05 | 0.00262727 |
| TCONS_01968613 | XLOC_997909 | 1728 | -0.951135 | 0.0001 | 0.00483418 |
| TCONS_01803508 | XLOC_898416 | 710 | -1.03668 | 0.00025 | 0.0100712 |
| TCONS_02523114 | XLOC_1223729 | 1749 | -1.22832 | 5.00E-05 | 0.00262727 |
| TCONS_00489746 | XLOC_238847 | 2548 | -1.32372 | 5.00E-05 | 0.00262727 |
| TCONS_02443383 | XLOC_1189568 | 1088 | -1.43686 | 0.0014 | 0.0401978 |
| TCONS_01645487 | XLOC_819639 | 1161 | -1.48264 | 5.00E-05 | 0.00262727 |
