# Supplementary material for: Expression status and clinical significance of lncRNA APPAT in the progression of atherosclerosis
Source: PeerJ. 2018 Jan 17;6:e4246. doi: 10.7717/peerj.4246 (PMC5775756; doi:10.7717/peerj.4246)
Supplement: Table S2 [file peerj-06-4246-s002.docx]

|  | **Transcript_id** | **Designed Primer** |
| --- | --- | --- |
| Rabbit | TCONS_02288701 | F: CCGTCCCTGTCAACACATCA  R:AGTGAGTCTAAGTGAGAACAGCTG |
| Rabbit | TCONS_02225105 | F: TTCCCAACCAACCAGCAAGT  R: GCACGCTAACTCTCTGGTGT |
| Rabbit | TCONS_00489746 | F: CCTGAAGCAATGGCATGAGC  R: ATCTGGCCTTCTCCTGGAGT |
| Rabbit | TCONS_02443383 | F: AGAGCCCACCATCTTCGTTG  R: CGCAAGAGGAGACACCAACT |
| Human | ENST00000551940 | F: GCCAGAAGCAGGAAGGTACA  R: CCACAGTGCCTAGAACAGCA |
| Human | ENST00000620272 | F: GCAGAGGCAGGTCACCAAC  R: CAGGATGATTCGAGACCAGGA |
| Human | ENST00000454551 | F: CTCAGAGACTTCCTCGGTTGT  R: TGACAGCATCCACACCCATC |
| Human | ENST00000526436 | F: TAGGCCTGAACCGTTTCACC  R: CCTCCTGCCTGACTCTCTCT |
