# Supplementary material for: Expression status and clinical significance of lncRNA APPAT in the progression of atherosclerosis
Source: PeerJ. 2018 Jan 17;6:e4246. doi: 10.7717/peerj.4246 (PMC5775756; doi:10.7717/peerj.4246)
Supplement: Table S3 [file peerj-06-4246-s003.docx]

| **Rabbit transcript ID** | **Human transcript name** | **Human Gene ID** | **Genomic Location**  **Of Human Gene** | **E-val** | **%ID** |
| --- | --- | --- | --- | --- | --- |
| TCONS_02443383 | [RP11-1028N23.4](http://asia.ensembl.org/Homo_sapiens/Gene/Summary?db=core;g=ENSG00000257407;tl=5QvMppw7vBTOK1NS-1628175-380496410) | ENST00000551940 | 12:115601840-115620150 | 6.00E-07 | [80.82](http://asia.ensembl.org/Homo_sapiens/Tools/Blast/Alignment?db=core;tl=5QvMppw7vBTOK1NS-1628175-380496410) |
| TCONS_00489746 | [AC159540.1](http://asia.ensembl.org/Homo_sapiens/Gene/Summary?db=core;g=ENSG00000230606;tl=SZFqPoLRhDmDCTaH-1633778-381889553)-017 | ENST00000620272 | 2:97426996-97427122 | 2.00E-11 | [82.68](http://asia.ensembl.org/Homo_sapiens/Tools/Blast/Alignment?db=core;tl=SZFqPoLRhDmDCTaH-1633778-381889553) |
| TCONS_02225105 | [RP13-210D15.4](http://asia.ensembl.org/Homo_sapiens/Gene/Summary?db=core;g=ENSG00000238210;tl=5QvMppw7vBTOK1NS-1628174-380496398) | ENST00000454551 | X:135253275-135253490 | 8.00E-11 | [62.78](http://asia.ensembl.org/Homo_sapiens/Tools/Blast/Alignment?db=core;tl=5QvMppw7vBTOK1NS-1628174-380496398) |
| TCONS_02288701 | [AP000479.1](http://asia.ensembl.org/Homo_sapiens/Gene/Summary?db=core;g=ENSG00000255433;tl=5QvMppw7vBTOK1NS-1628172-380496662) | ENST00000526436 | 11:56876268-56876333 | 4.00E-04 | 76.81 |
