# Supplementary material for: Expression status and clinical significance of lncRNA APPAT in the progression of atherosclerosis
Source: PeerJ. 2018 Jan 17;6:e4246. doi: 10.7717/peerj.4246 (PMC5775756; doi:10.7717/peerj.4246)
Supplement: Table S4 [file peerj-06-4246-s004.docx]

| **Num.** | **Sequence** |
| --- | --- |
| 1 | 5’-GTTTCCTGTTCCTGTGTTAATTAGCTGAAGTTTATGGCTTCCAGCTTCATCCATGTCCCTGCAAAAGACATGATCTCATT-3’ |
| 2 | 5’-CCTTTTTATGGCTGCATAGTATTCCATGGTGTATATGTATCACATTTTCTTTATCCAGTCTATCATTGATGGACATTTGG-3’ |
| 3 | 5’-GCTGATTCCATGTCTTTGCTATTGTGAATAGTGCTCAGAATTTGCTTGTCTGTAAATGATCTCATTTCTCCTTCATTTAG-3’ |
