# Supplementary material for: Expression status and clinical significance of lncRNA APPAT in the progression of atherosclerosis
Source: PeerJ. 2018 Jan 17;6:e4246. doi: 10.7717/peerj.4246 (PMC5775756; doi:10.7717/peerj.4246)
Supplement: Table S5 [file peerj-06-4246-s005.docx]

| **miRNA** | **has-miR-647** | **has miR-135b** | **has-miR-1229** |
| --- | --- | --- | --- |
| Transcript position | 407-423 | 352-374 | 207-226 |
| Position on chromosome | 2:97427022-97427039 | 2:97426967-97426990 | 2:97423252-97423272 |
| Conserved species | panTro2 | panTro2 | panTro2, rheMac2, danRer6 |
| Binding area | 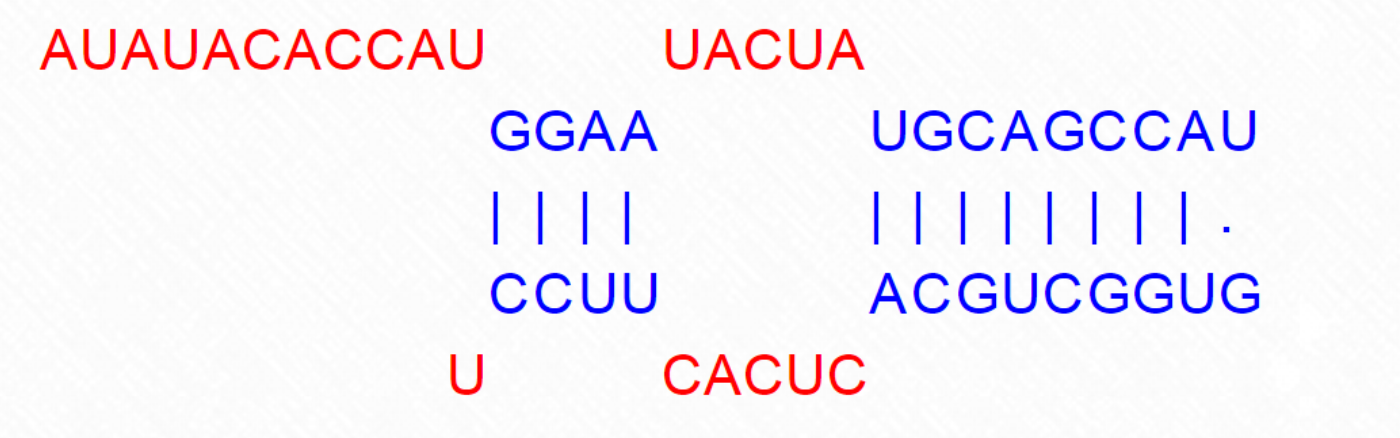 | 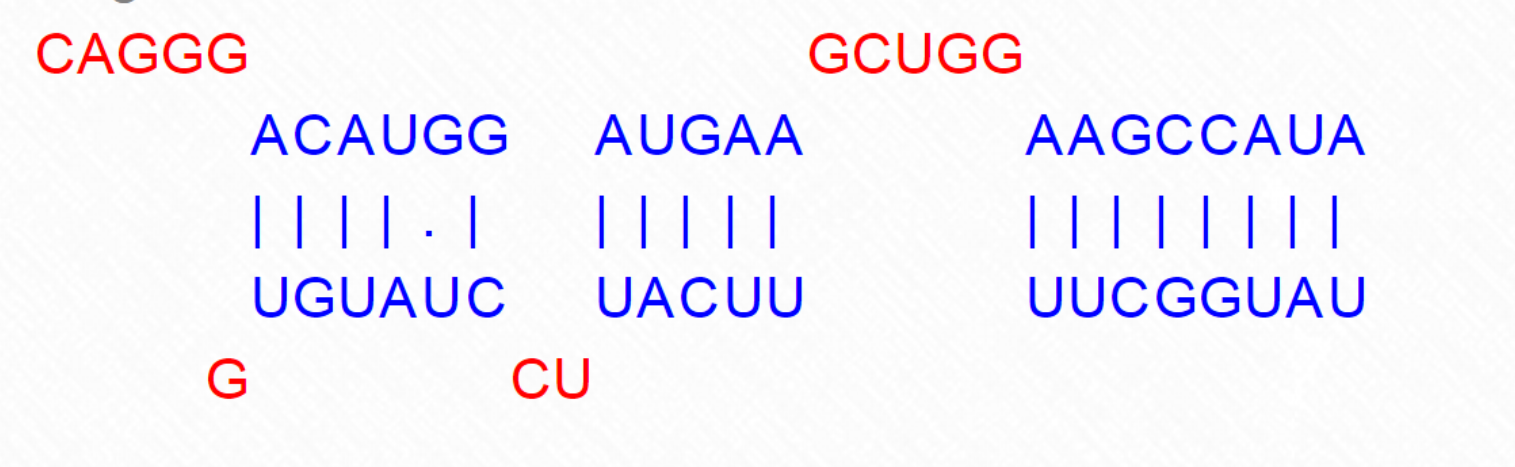 | 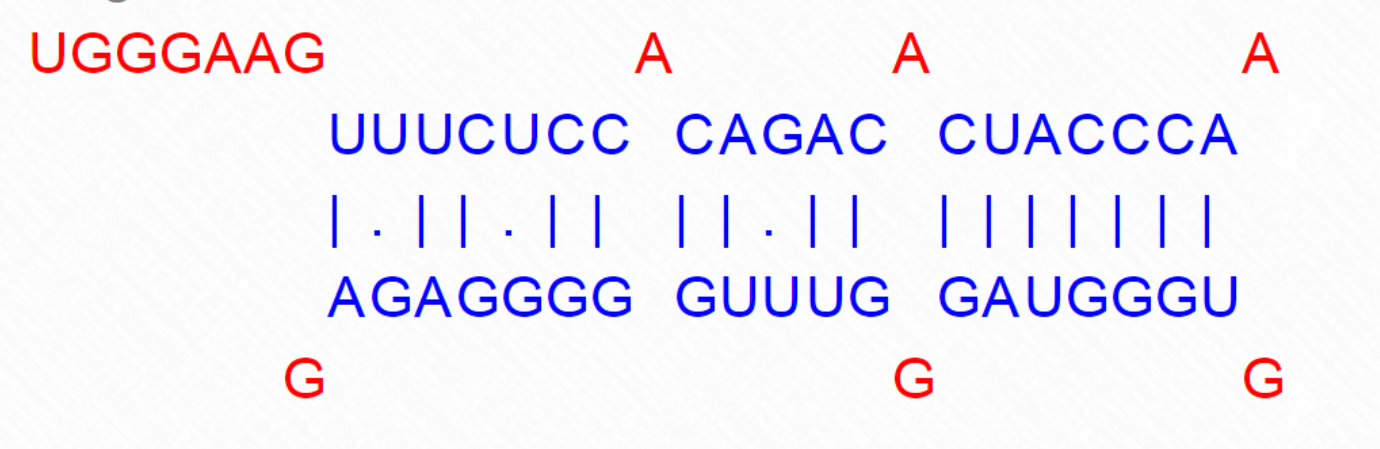 |
