# Supplementary figures and images for: Expression status and clinical significance of lncRNA APPAT in the progression of atherosclerosis
Source: PeerJ. 2018 Jan 17;6:e4246. doi: 10.7717/peerj.4246 (PMC5775756; doi:10.7717/peerj.4246)

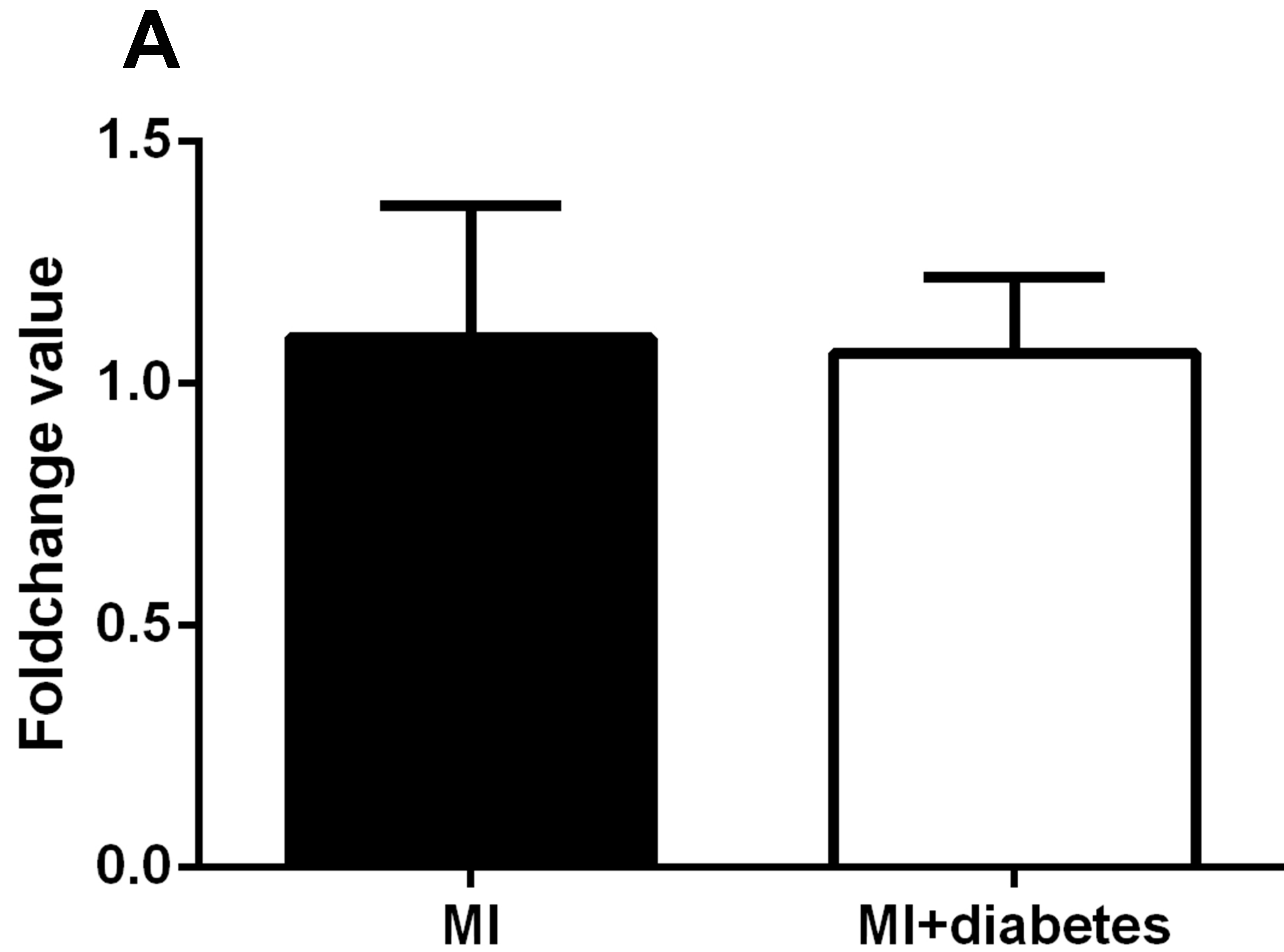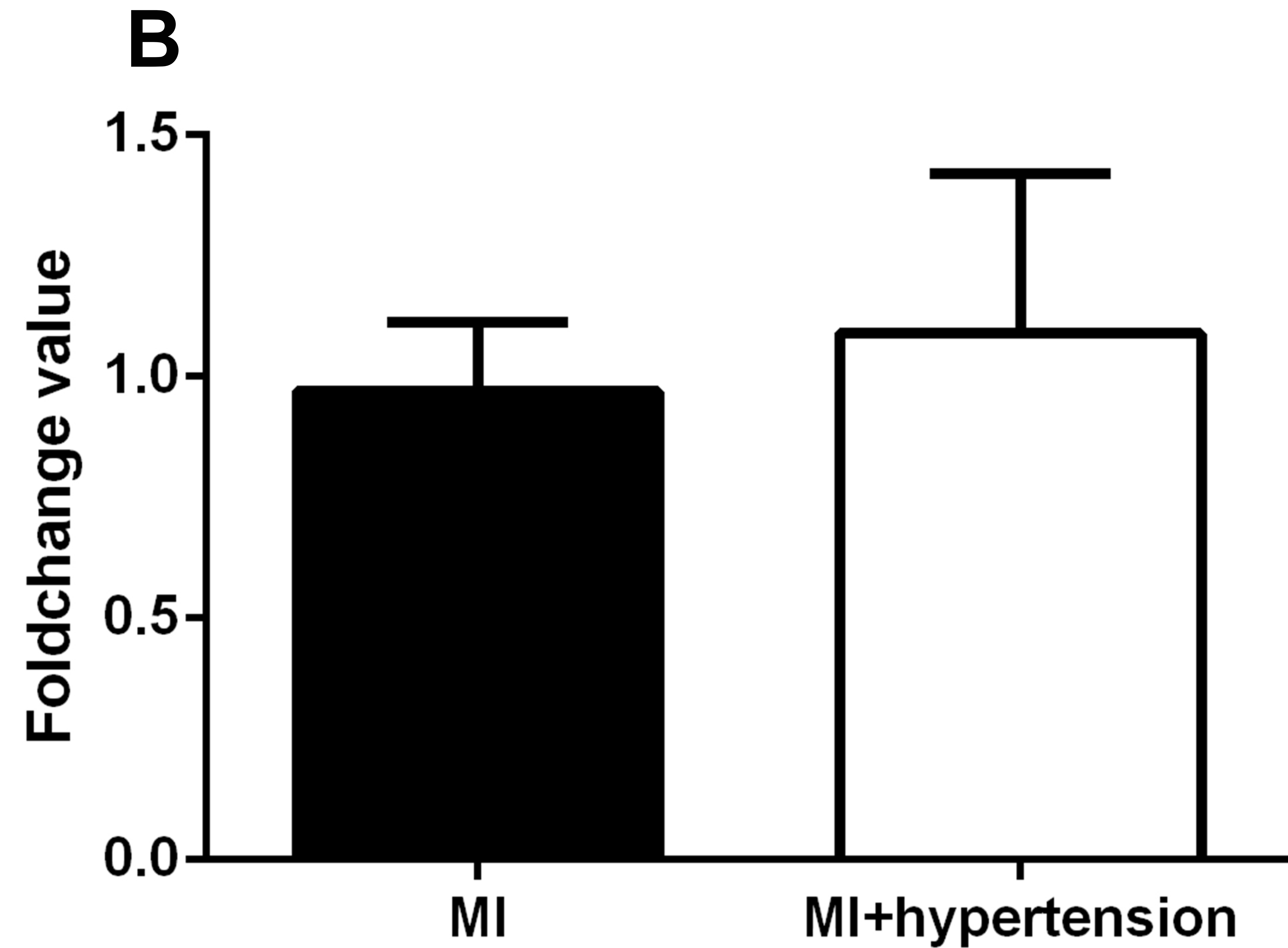

Supplement: Figure S1 [file peerj-06-4246-s006.pdf]

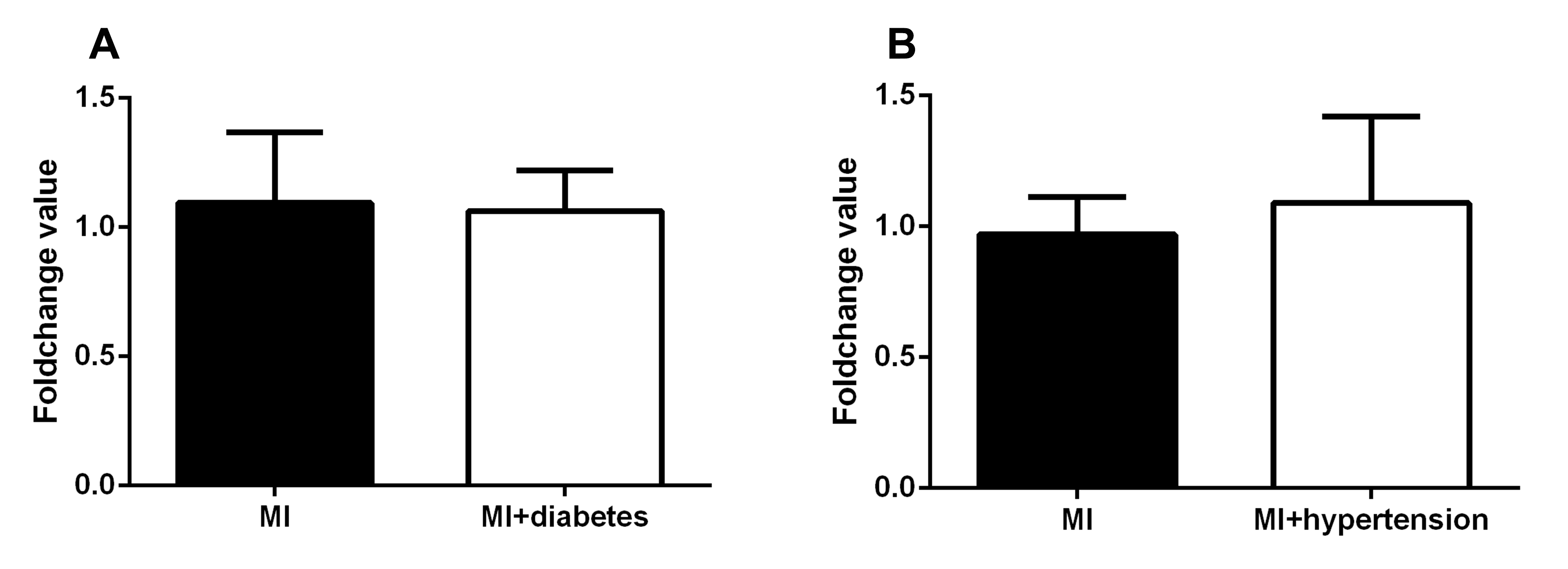

Supplement: Figure S2 [file peerj-06-4246-s007.png]
